# New high-Z detectors based on YAlO_3_:Bi^3+^ for joint use with tissue equivalent BeO in tandem OSL dosimeter

**DOI:** 10.1038/s41598-025-05285-6

**Published:** 2025-07-01

**Authors:** Yaroslav Zhydachevskyy, Vasyl Stasiv, Oleksandr Poshyvak, Sergii Ubizskii, Zuzanna Pawłowska, Yuliia Rumiantseva, Magdalena Baran, Halyna Zhydachevska, Marek Berkowski, Andrzej Suchocki

**Affiliations:** 1 Institute of Physics, Polish Academy of Sciences, aleja Lotników 32/46, 02-668 Warsaw, Poland; 2 Berdyansk State Pedagogical University, Shmidta Str. 4, Berdyansk, 71100 Ukraine; 3 Lviv Polytechnic National University, S. Bandera Str. 12, Lviv, 79013 Ukraine; 4 Central Laboratory for Radiological Protection, Konwaliowa 7, 03-194 Warsaw, Poland; 5 Łukasiewicz Research Network–Krakow Institute of Technology, Zakopianska 73, 30-418 Kraków, Poland; 6 Łukasiewicz Research Network–Institute of Microelectronics and Photonics, Wólczyńska 133, 01-919 Warsaw, Poland

**Keywords:** Optically stimulated luminescence, OSL dosimetry, High-Z detectors, YAP:Bi, BeO, Engineering, Materials science, Optics and photonics

## Abstract

The work introduces a new type of UV-emitting high-*Z* OSL detectors based on YAlO_3_:Bi^3+^ (YAP: Bi) perovskite. Two kinds of YAP: Bi solid state detectors have been fabricated and compared to BeO Thermalox^®^995 chips. The first ones are single-crystalline detectors cut from a Czochralski grown crystal. The second are detectors cut from the high-density bulk ceramics prepared by the high-pressure high-temperature (HPHT) pressing technique from nanocrystalline powder derived from sol-gel synthesis. The YAP: Bi ceramic detectors studied suffer from strong thermal fading, which makes their use impractical, at least until technological methods to modify this property of ceramics are found. The single crystalline YAP: Bi detectors show very low thermal fading, a wide linearity range of dose response and a sensitivity comparable to BeO, which together with the optical registration in the same UV range, compatible optical stimulation by blue light and similar registration times in CW-OSL mode make them ideal high-*Z* detectors that can be used alone or in tandem with BeO detectors.

## Introduction

Thermally (TSL) and optically stimulated luminescence (OSL) are well-known phenomena widely used in radiation dosimetry^1–5^. The OSL technique, which has several advantages over TSL, has become increasingly popular in the last decade for personal and medical dosimetry^6–8^. Despite the large number of works devoted to the study of already known and new storage phosphors applicable to OSL dosimetry (see e.g., Refs^9–20^. and the recent reviews^8,21,22^), only two of them, namely Al_2_O_3_:C^23^ and BeO^24^ are used practically in commercial dosimetric systems.

Tissue equivalence of dosimetric phosphors is one of the most important requirements for dosimetric materials used in personal and medical dosimetry. This equivalence means the similarity of the radiation energy absorption in the dosimetric detector and living tissue, and thus the similarity of the interaction processes of radiation of different energy and nature in both materials, which is ensured by the similarity of their effective atomic *Z*_*eff*_ numbers. This circumstance has led to the fact that out of a large number of all known phosphors that have been investigated as candidates for TSL/OSL dosimetry, only a small number (see, e.g., Yukihara et al.^8^) have an effective atomic number significantly higher than the *Z*_*eff*_ of living tissue ~ 7.5^25^. Nevertheless, materials with a high atomic number and thus a much higher sensitivity of their luminescence response in the low photon energy range (< 500 keV) are also in demand and are of particular interest to researchers in X-ray imaging^26–29^. One of the few high-*Z* materials that has been relatively well studied for potential use in TSL/OSL dosimetry is YAP: Mn^2+^ with *Z*_*eff*_ = 31.4^30,31^, which has demonstrated a number of its advantages in TSL dosimetry^30–32^, but has revealed some properties that limit its use in OSL dosimetry^33^.

On the other hand, the difference in the energy dependence of the luminescence response of storage phosphors with different *Z*_*eff*_ potentially allows the recognition of radiation by its spectral composition^34^ when used in tandem in one dosimeter^35^. This old idea has been returned to many times^36–39^, and recently, it has been used^40,41^ in the study of the feasibility of recognizing radiation from unknown radioisotopes when using two dosimetric OSL phosphors, BeO (*Z*_*eff*_ = 7.2^24,42^) and YAP: Mn (*Z*_*eff*_ = 31.4), in tandem. This problem was considered an urgent task in the context of in emergency dosimetry to ensure preparedness for a terrorist radiation attack using unknown in advance radiation sources.

YAlO_3_:Mn^2+^ detectors have been shown to be quite efficient and suitable for this purpose, having a 40-fold increase in the sensitivity at 45 keV compared to 1 MeV^31^. However, as OSL detectors, they have some drawbacks, such as long afterglow, and emission in the visible range, which is affected by the photoluminescence of Mn^2+^, requiring a special and complex OSL readout^33,43^. This is particularly inconvenient when it should be used alongside UVemitting OSL phosphors such as BeO or Al_2_O_3_:C. Therefore, our challenge was to search for a new efficient UVemitting high-Z OSL phosphor that has none of the drawbacks of YAP: Mn, but possesses main advantages of its matrix.

YAlO_3_ (YAP) perovskite, especially in the form of single crystals, has confirmed its suitability and efficiency as a high-*Z* host for TSL/OSL phosphors, offering the following advantages: (i) an extremely wide range of linearity of dose dependence (from a few mGy to a few kGy), and (ii) very high mechanical, chemical and thermal stability, (iii) high resistance to the radiation damage^30–32^. Thinking about another (alternative to Mn^2+^) activation of the YAP host that would allow emission in the UV range, we turned our attention to bismuth (Bi^3+^) doping. As shown previously, the intracenter emission of Bi^3+^ ions occupying Y sites in the YAP host is around 325 nm^44,45^. Also, Bi^3+^ ions in YAP and other oxide crystalline hosts under ionizing irradiation are ionized to Bi^4+^ by trapping the released electrons on electron traps available in the host^45,46^. The TSL glow observed in YAP: Bi^3+^ mainly in a peak at about 200 °C is suggested to occur as a result of the release of electrons from the electron centers intrinsic to the YAlO_3_ lattice and their recombination with the Bi^4+^ hole centers^45^. It should be noted that the TSL properties of YAP: Mn^2+^ crystals above room temperature are mainly determined by the similar ($\:{\text{M}\text{n}}_{\text{Y}}^{2+}{\text{M}\text{n}}_{\text{Y}}^{3+}$ + *e*) recharging of manganese ions under ionizing radiation^47^ and the presence of intrinsic electron traps of the YAP host, which by the way are responsible for the main TSL peak at about 200 °C^30,48^. Thus, combining of Bi^3+^ doping and YAP host can be very attractive in obtaining the TSL/OSL properties with the UV emission required for radiation dosimetry. Therefore, we aimed to prepare YAP: Bi^3+^ detectors and to explore their TSL/OSL dosimetric properties, especially with respect to YAP: Mn^2+^ detectors, as well as to compare them with the well-known UV-emitting low-*Z* detectors, such as BeO ceramics, with which they can be used in tandem.

Two different methods were used to prepare high-density bulk YAP: Bi^3+^ detectors. The first one was an attempt to grow YAP: Bi^3+^ crystals using the conventional Czochralski technique. The second was the ceramic prepared by the high-pressure high-temperature (HPHT) pressing of nanocrystalline powders derived from sol-gel synthesis. The TSL and OSL properties of both types of YAP: Bi^3+^ detectors have been studied in detail and compared both with studied previously YAP: Mn^2+^ crystalline detectors and with the commercially available BeO (Thermalox^®^995) ceramic detectors.

## Materials and methods

### Crystals growth and ceramic samples preparation

**Fig. 1:**
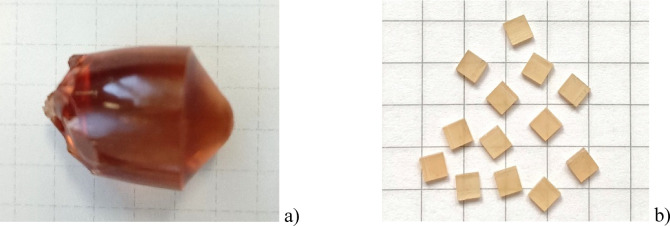
Single crystal of YAP: Bi (< 0.1%) grown by the Czochralski technique (**a**) and the crystalline 3 × 3 × 1 mm^3^ chips (**b**) cut from the top of the boule.

The Bi^3+^-doped YAP crystals were grown at the Institute of Physics of the Polish Academy of Sciences by the Czochralski technique as described in Ref^49^. Several growth experiments were performed, which confirm the high evaporation rate of bismuth oxide during melt homogenization and crystal growth processes. The high evaporation rate of bismuth oxide is undoubtedly the main technical difficulty in growing YAP crystals with the desired concentration of Bi^3+^ ions. Utilization of the excessive concentration of bismuth in the raw materials (up to 5%) together with the reduction of the melt homogenization time allowed to grow a crystal as shown in Fig. 1 with a relatively low concentration of Bi^3+^ ions, which we estimate from the photoluminescence intensity to be less than 0.1% with respect to yttrium. The crystalline YAP: Bi detectors studied here were cut as 3 × 3 × 1 mm^3^ chips of the 50 ± 2 mg mass from one 3 mm thick radial plate cut from the top of the boule normal to the direction of crystal growth. It should be noted that the as-grown YAP: Bi crystals have a reddish color that is typical for YAP crystals and is caused by intrinsic color centers^50,51^.

**Fig. 2:**
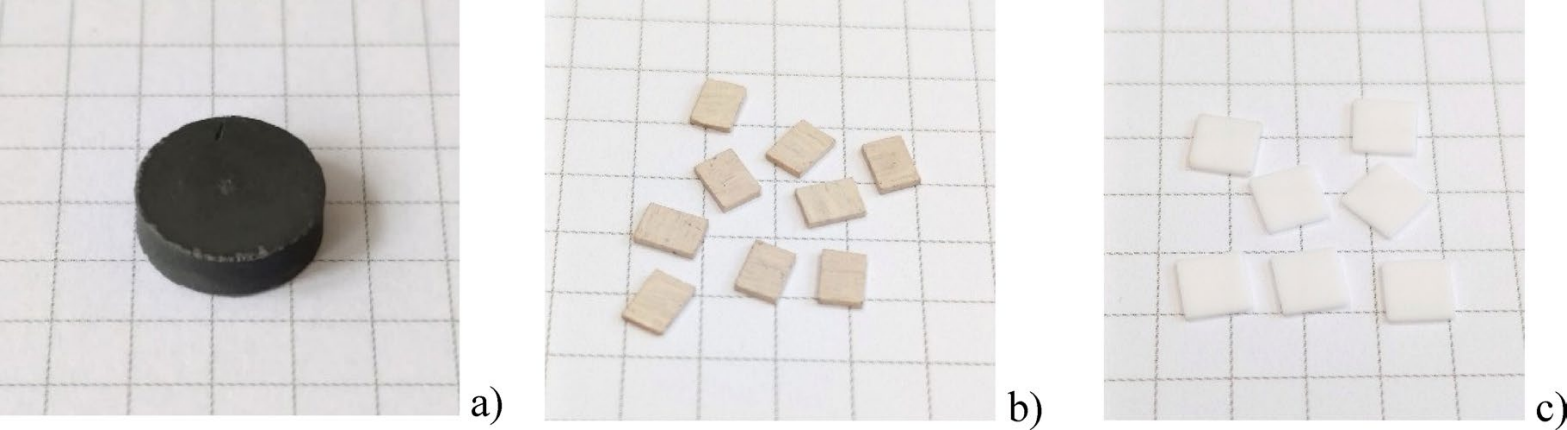
One pellet of HPHT YAP: Bi ceramics (**a**) and the 4 × 3 × 0.5 mm^3^ YAP: Bi ceramic chips (**b**) cut from the pellet, in comparison with the BeO Thermalox^®^995 chips (**c**).

In addition to single crystals, nanocrystalline powders of YAP: Bi were synthesized by the sol-gel technique as it was described previously by Krasnikov et al.^44^ An advantage of the wet chemical synthesis of YAP: Bi is that it is possible to avoid bismuth evaporation and thus obtain the material with the desired concentration of Bi^3+^ ions, but only under the condition of thermal treatment at temperatures not higher than about 1200 °C^45^. For the purposes of this work, the YAP: Bi(2%) nanopowders have been synthesized and calcined at 900 °C. This powder was then processed into high-density solid ceramic pellets by the high-pressure high-temperature (HPHT) pressing technique using a Model DO OO4 Hybrid HPHT-SPS apparatus equipped with a Bridgman chamber using a conventional resistive heating at 50 Hz altering current^52^. The typical size of the pellets was 12 mm in diameter and approximately 4 mm thick. Typical thermobaric parameters for HPHT sintering were a temperature of 500 °C and a pressure of 7.0 GPa for 3 minutes. It was expected that high pressure would allow theoretical density values to be achieved and internal porosity to be minimized, thereby improving the transparency of the sintered samples even at relatively low sintering temperatures, similar to the results reported by Yavetskiy et al.^53^ The pellets as prepared were usually dark grey or black on the outside and less grey or white on the inside, as shown in Fig. 2, due to carbon incorporation from the graphite crucible. From one such a pellet, approximately 20 pieces of the 4 × 3 × 0.5 mm^3^ ceramic chips can be cut. After thermal annealing of the chips in air at 1200 °C, the grey coloration disappeared and the chips became a light reddish color like a single crystal from the Czochralski growth. The phase purity of the YAP: Bi ceramics under investigation was confirmed by powder X-ray diffraction studies and by the Bi^3+^ emission features shown below. Even after the high-temperature annealing in air, the YAP: Bi ceramic chips remained opaque to visible light. The mass of 34 ± 2 mg of the 4 × 3 × 0.5 mm^3^ chips confirms that the density of the studied ceramics is very close to the 100% density of the material, which is 5.35 g/cm^3^^54^.

The BeO Thermalox^®^995 translucent chips of about 5 × 5 × 0.5 mm^3^, which will be further referred to as BeO ceramics, were supplied by RadPro International GmbH and used by us as reference detectors.

### Characterization

All the luminescence (PL, RL, TSL and OSL) measurements were done using the registration channel of a Horiba/Jobin-Yvon Fluorolog-3 spectrofluorometer equipped with a Hamamatsu R928P photomultiplier operating in a photon counting mode. The photoluminescence (PL) and radioluminescence (RL) spectra were corrected for the spectral response of the used system. For the OSL measurements, an additional bandpass fluorescence filter (340 nm CWL, 26 nm bandwidth, OD6) was used. The TSL measurements were done using a Linkam THMS600 temperature stage. The continuous wave optically stimulated luminescence (CW-OSL) measurements were done using a blue laser diode (450 nm, 0.7 W optical power) for optical stimulation. The laser beam was defocused by a lens to cover the entire sample under investigation. The CW-OSL curves were measured in real time with the same spectrometer using the 9.5 nm spectral bandwidth at 325 nm and the 1 ms discrimination time.

The X-ray irradiation of the samples was done using a 130 kV microfocus L9181-02 Xray source with the sample placed at a distance of 50 mm from the Be window of the source. The control exposure to Cs-137 was done using the facilities of the Central Laboratory for Radiological Protection, Poland.

After exposure to ionizing radiation, detectors were normally stored in the dark (wrapped in aluminum foil) unless otherwise specified.

## Results and discussion

### Basic emission properties

**Fig. 3:**
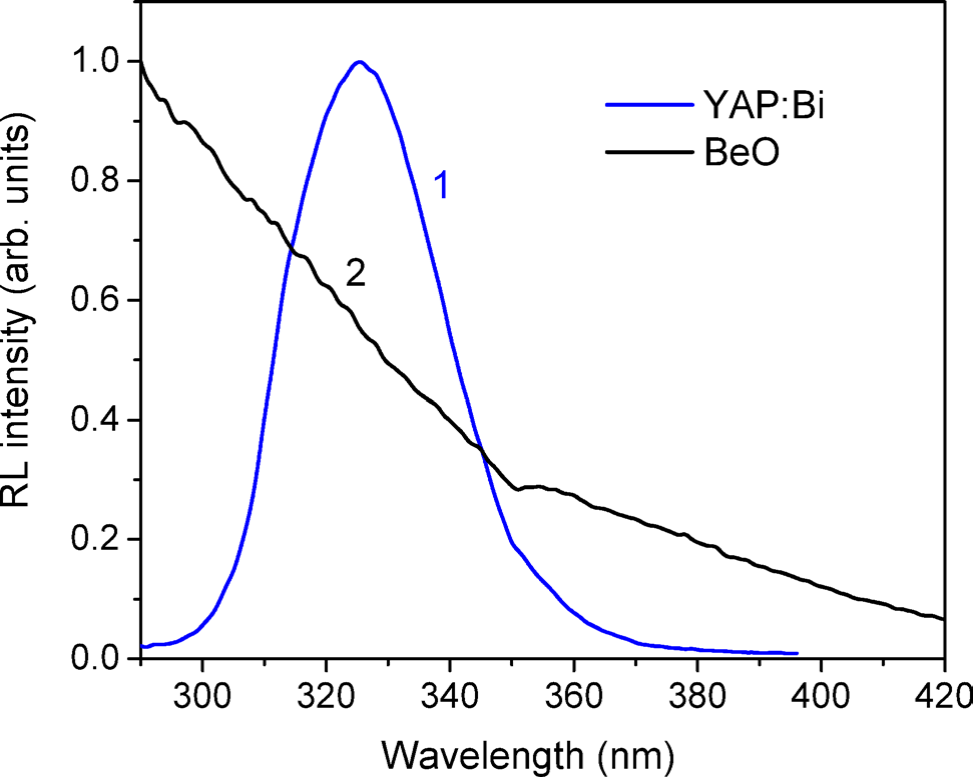
Normalized radioluminescence spectra of ceramic YAP: Bi (1) and BeO (2) detectors measured at continuous X-ray (130 kV) excitation at room temperature.

Typical radioluminescence spectrum of the YAP: Bi ceramic samples under continuous Xray (130 kV) excitation is shown in Fig. 3. The same emission band at about 325 nm, caused by the intracenter emission of Bi^3+^ ion in YAP, is also observed in PL at about 270 nm excitation as well as in TSL and OSL^45^. It should be noted that the crystalline YAP: Bi samples showed at least one order of magnitude lower PL and RL emission intensity compared to the ceramic samples evidently due to the much lower concentration of Bi in the crystal under investigation. The RL spectrum of BeO ceramics is also shown in Fig. 3 for comparison. As reported previously, the RL spectrum of BeO ceramics extends far into the UV^55^, while the OSL emission occurs in a broad band around 325 nm^56^.

**Fig. 4:**
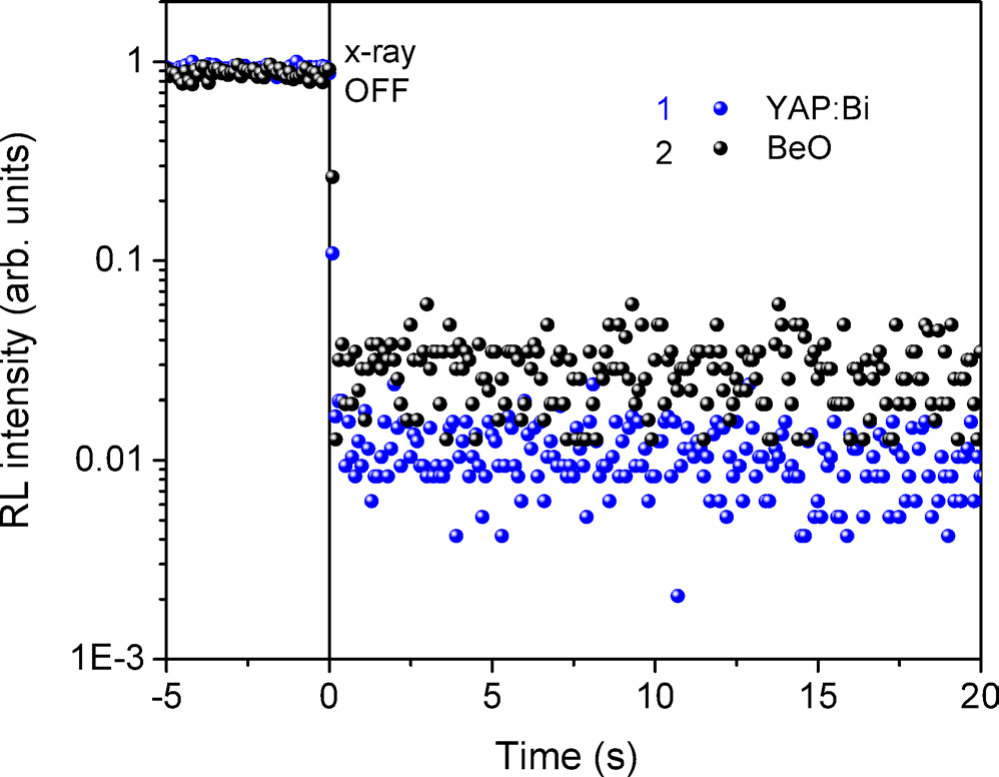
Check for afterglow of the ceramic YAP: Bi (1) and BeO (2) detectors after X-ray excitation, optical registration at 325 nm.

Considering that the significant afterglow of the crystalline YAP: Mn detectors was previously shown by Ubizskii et al.,^33^ we were interested to see how it would be in the case of YAP: Bi. Our measurements, shown in Fig. 4, show no evidence of afterglow in both YAP: Bi and BeO detectors or it lasts much less than 1s. Due to the much lower RL intensity of the crystalline YAP: Bi samples, we were unable to reveal afterglow.

### TSL and OSL properties

**Fig. 5:**
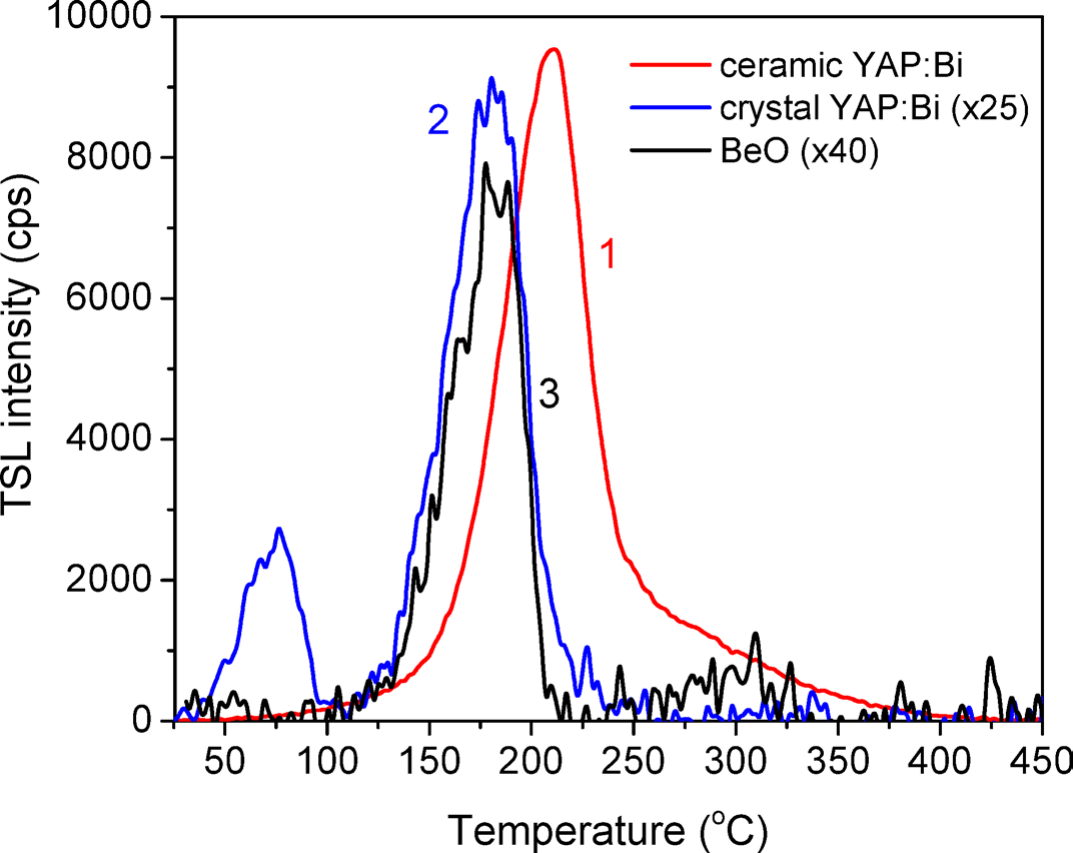
Thermal glow curves of the ceramic YAP: Bi (1), crystalline YAP: Bi (2) and BeO (3) detectors, registered at 325 nm with a heating rate 2 °C/s immediately after X-ray exposure at room temperature.

Typical thermal glow curves of the studied detectors registered in the same exposure and measuring conditions are presented in Fig. 5. Since the 130 kV X-ray exposure was used here, the TSL signal of BeO was multiplied by 40 to compensate for the 40-fold overresponse of YAP over BeO for the radiation energies around 45 keV^31^. As seen from the Fig. 5, the ceramic YAP: Bi and BeO detectors of comparable dimensions show in such a way a comparable level of TSL output. The crystalline YAP: Bi detectors in its turn show about 25 times less TSL output. It is also noticeable that the main TSL peak for the crystalline YAP: Bi has the maximum shifted slightly towards lower temperatures compared to the ceramic YAP: Bi. In addition to the main TSL peak at about 200 °C, the ceramic YAP: Bi detectors reveal a structureless higher temperature peak at about 250–300 °C, which does not occur for the crystalline YAP: Bi. At the same time, the crystalline YAP: Bi detectors also have a lower temperature peak at around 75 °C.

**Fig. 6:**
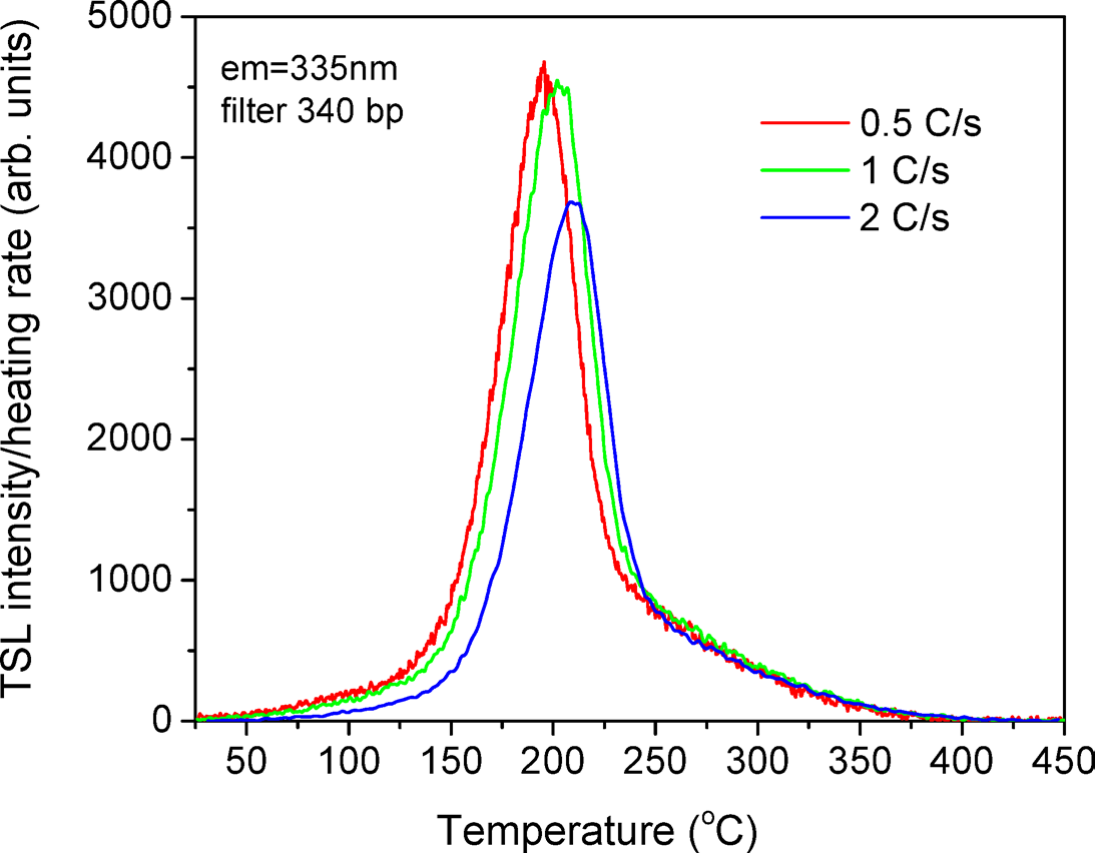
Thermal glow curves of the ceramic YAP: Bi detectors registered immediately after Xray exposure at room temperature at different heating rates as shown.

The thermal glow curves of the ceramic YAP: Bi detectors measured at different heating rates (Fig. 6) show that the TSL output decreases as the heating rate increases. This should suggest that the Bi^3+^ related emission in YAP undergo thermal quenching. In fact, previous photoluminescence studies have shown that thermal quenching of Bi^3+^ UV emission in YAP starts at temperatures above 200 K (–73 °C)^45^. Therefore, similar to BeO or Al_2_O_3_:C, which undergo strong emission intensity quenching at elevated temperatures, one can expect that YAP: Bi should be more efficient and convenient in OSL.

**Fig. 7:**
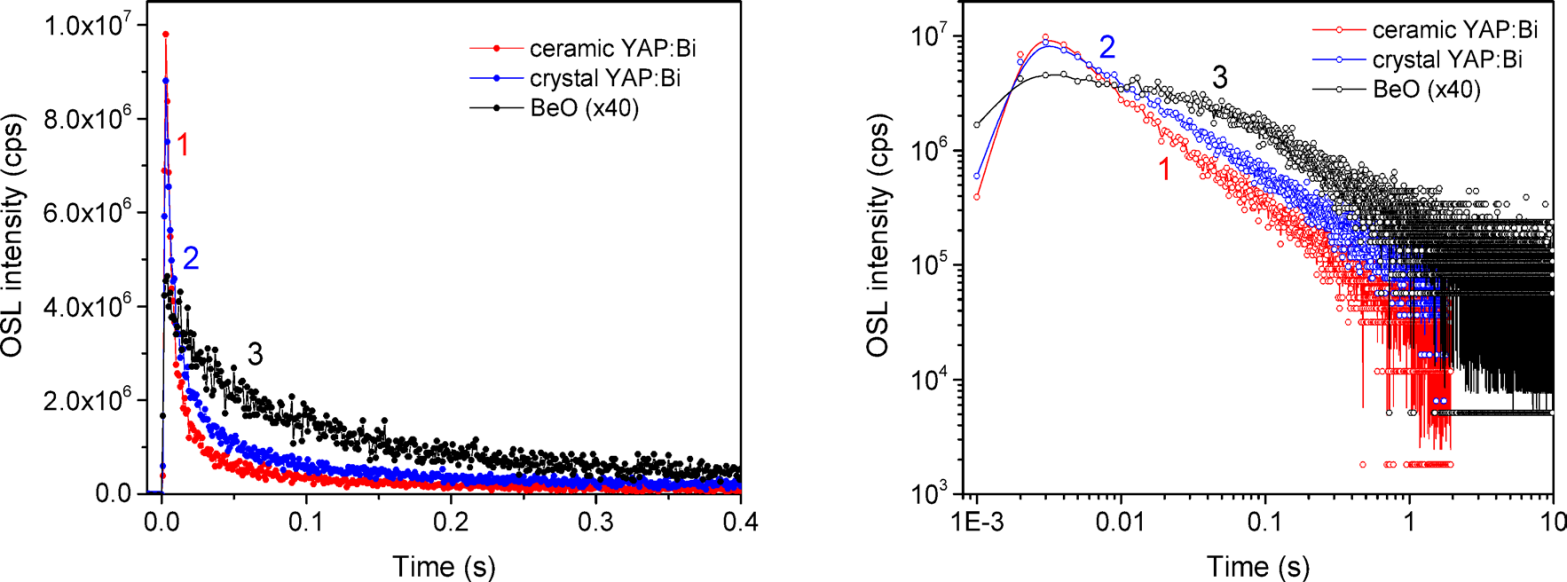
CW-OSL curves of the ceramic YAP: Bi (1), crystalline YAP: Bi (2) and BeO (3) detectors registered at 335 nm under blue laser (450 nm, 0.7 W) optical stimulation immediately after X-ray exposure (130 kV, 30 µA, 600 s).

**Fig. 8:**
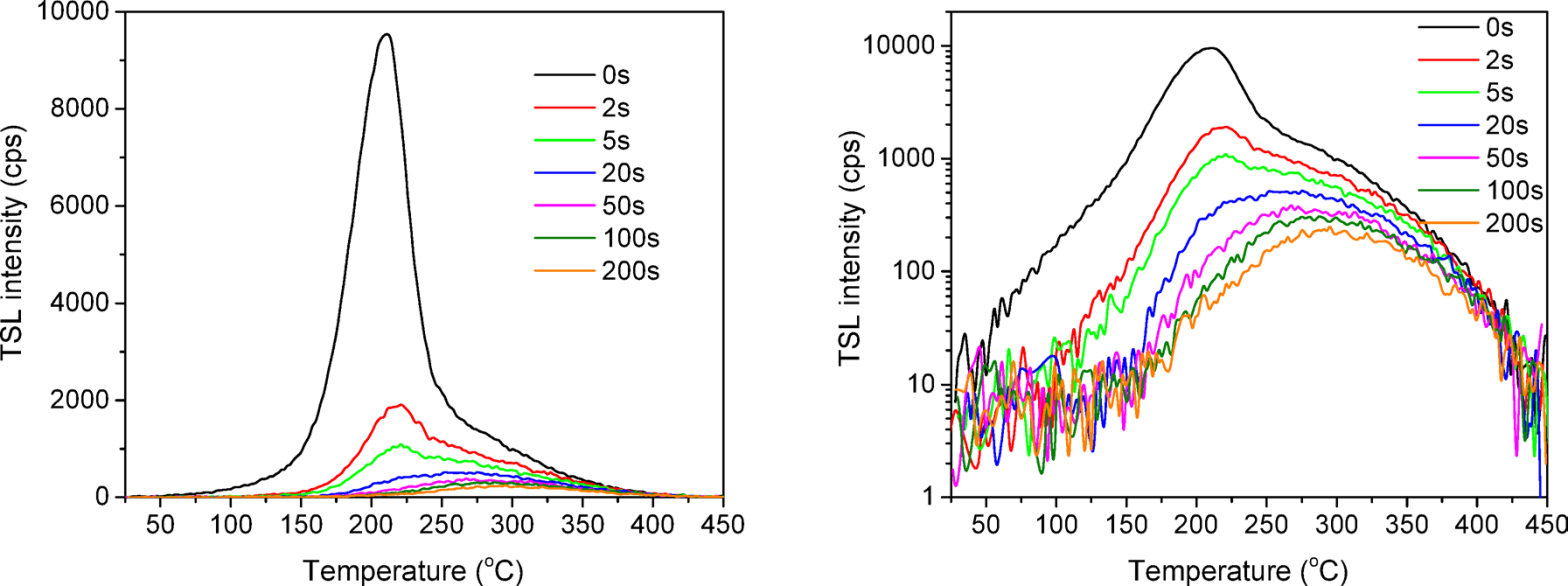
The effect of optical stimulation by the blue laser (450 nm, 0.7 W, the stimulation duration is indicated) on the residual TSL of the ceramic YAP: Bi detectors after X-ray exposure. The left and right plots show the same data plotted in the linear and logarithmic ordinate scales, respectively.

Typical OSL curves of the studied detectors under continuous blue laser stimulation are shown in Fig. 7. As one can see from the Fig. 7, OSL decay of both ceramic and crystalline YAP: Bi detectors occurs essentially faster than for BeO, showing a higher intensity at the initial point in time. The higher initial OSL intensity and a faster decay of YAP: Bi over BeO suggest more efficient optical stimulation, i.e. release of the trapped carriers under the same optical stimulation. The higher initial OSL intensity should also mean greater reliability in measuring smaller doses with YAP: Bi detectors. Similarly, as for TSL, the OSL output of BeO after X-ray exposure was multiplied here by 40 to straighten BeO and YAP out in the energy response. The crystalline YAP: Bi detectors show a comparable initial intensity and a slightly longer OSL decay (at least up to 1 s of stimulation) compared to the ceramic YAP: Bi detectors. Taking the OSL intensity integrated in time from 0 to 1 s as a criterion, the ratio is approximately 1:1.5:3 for ceramic YAP: Bi, crystalline YAP: Bi and BeO, respectively. The one and a half times higher OSL output of the crystalline YAP: Bi compared to the ceramic YAP: Bi is quite unexpected, knowing that the TSL output of the former is about 25 times lower (see Fig. 5). This observation becomes clear when analyze the residual TSL signal after the optical stimulation used. As seen from Fig. 8, even 200 s of the stimulation is not enough to bleach completely the ceramic YAP: Bi, while for the crystalline YAP: Bi, we were unable to register any residual TSL already after 2 s of such stimulation. In other words the crystalline YAP: Bi releases all the accumulated lightsum during the short time of optical stimulation, while the ceramic YAP: Bi requires much longer stimulation time to release all the lightsum. This is most probably related with the high optical transparency of the crystalline YAP: Bi compared to the non-transparent YAP: Bi ceramics.

**Fig. 9:**
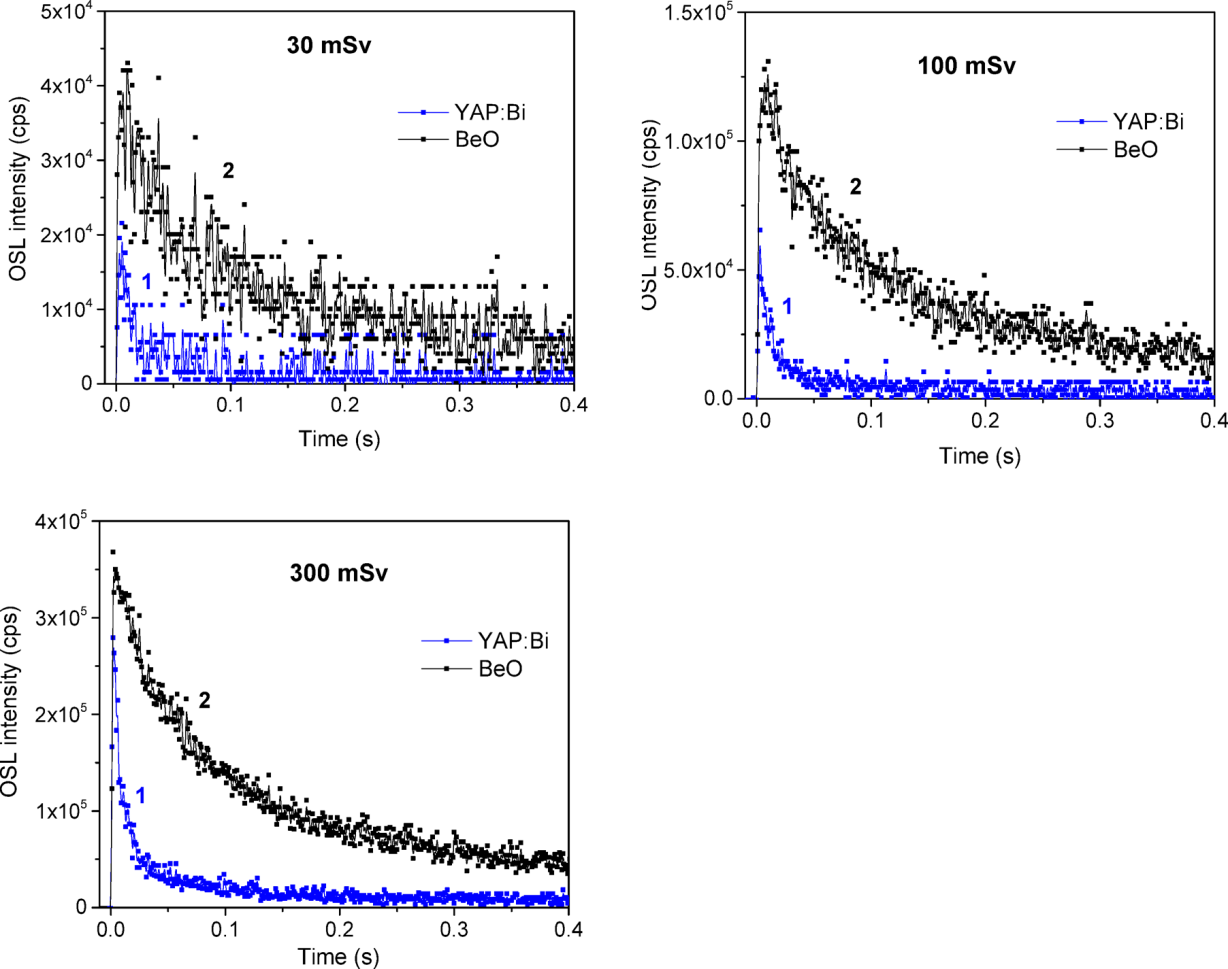
CW-OSL curves of the crystalline YAP: Bi (1) and BeO (2) detectors registered at 335 nm under blue laser (450 nm, 0.7 W) optical stimulation after Cs-137 exposure to doses 30, 100 and 300 mSv.

Figure 9 compares the CW-OSL output of the crystalline YAP: Bi and BeO detectors after low doses of gamma irradiation from a Cs-137 source. The relative energy response of YAP to this type of irradiation (energy 0.662 MeV) is close to 1.0^31^, therefore no additional scaling is required to directly compare the OSL output of YAP: Bi and BeO detectors. These results after gamma irradiation confirm a faster CW-OSL decay for the crystalline YAP: Bi when compared to BeO. However the initial OSL intensity here for the crystalline YAP: Bi detectors is somewhat lower than for BeO detectors, which in combination with the faster decay of YAP: Bi compared to BeO results in about 6 times lower OSL output when integrated in the 0–1 s time range. Knowing that the detection threshold of BeO ceramics is about 20 µGy^56^, it can be expected that the detection threshold of the crystalline YAP: Bi detectors studied here will not be much worse, especially due to a faster OSL decay, which should be easier to register against the background of a dark signal. To address this, separate studies of YAP: Bi detectors are required using the optical detection system, which is more sensitive in the UV than we have used here. The main properties of the YAP: Bi detectors studied are summarized in Table 1.

**Table 1:** The main properties of the YAP: Bi detectors studied and their qualitative comparison with BeO ceramics and single crystalline YAP: mn.

	YAP: Bi (nanoceramic)	YAP: Bi (crystal)	YAP: Mn (crystal)^30–32^	BeO (Thermalox^®^995)^56^
*Z*_*eff*_	31.4	31.4	31.4	7.2
Optical transparency	Opaque	Highly transparent	Highly transparent	Translucent
Main TSL peak (°C)	210	180	200	180
Emission wavelengths (nm)	325	325	530	≤ 420
Relative TSL output after Xray exposure	~ 2	~ 0.05	> 200	1 (×40)*
Afterglow	No	Not revealed	Yes^#^	No
Optical stimulation wavelengths (nm)	~ 450	~ 450	~ 525^32^ ~ 470^33^	~ 450
CW-OSL mode	Yes	Yes	No^#^	Yes
PL under optical stimulation	No	No	Yes^#^	No
Relative OSL output after Xray (130 kV) exposure	0.33	0.5	Not compared	1 (×40)*
The OSL decay rate relative to BeO	Faster	Faster	Not compared	–
Thermal fading	80%/24 hours	10%/3 months	≤ 20%/year	10%/3 months
Linearity of the OSL dose response	Not measured	Up to 10^3^ Sv	Up to 2⋅10^3^ Gy	Up to 10^2^ Gy

*See text for details.

^#^Main drawbacks of YAP: Mn OSL detectors.

### Thermal fading and dose response

**Fig. 10:**
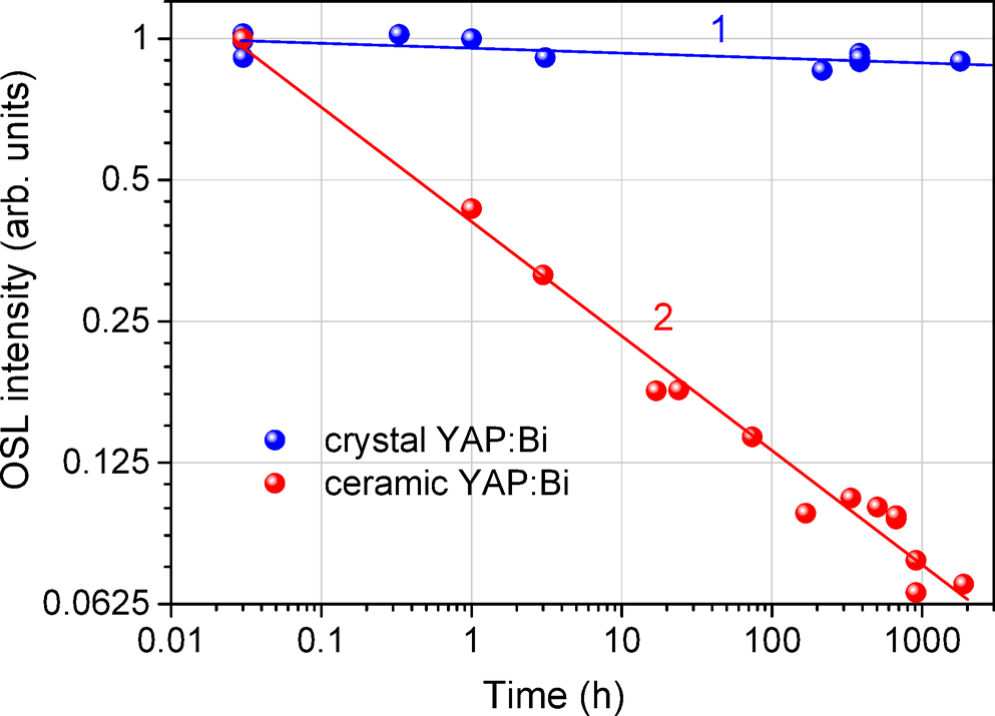
Thermal fading of the OSL signal of the crystalline YAP: Bi (1) and ceramic YAP: Bi (2) detectors during dark storage at room temperature. The solid lines represent the linear fit of the experimental points.

Our measurements revealed that YAP: Bi ceramics possess a considerable thermal fading during dark storage at room temperature. As one can see from Fig. 10, the ceramic YAP: Bi detectors lose almost 80% of the OSL signal during the first 24 h after exposure, so that only about 8% of the signal remains after one month of dark storage, that is similar to the fading of YAP: Mn nanocrystalline ceramics reported previously^57^. On the other hand, the results for the crystalline YAP: Bi detectors presented in Fig. 10 confirm the low fading not exceeding 10% in 3 months, which is consistent with ≤ 20%/year reported previously for the same TSL peak of YAP: Mn single crystals^30^. It should be noted that due to the low temperature TSL peak at about 75 °C for crystalline YAP: Bi, the detectors were preheated to 100 °C before recording the CW-OSL. We have experimentally confirmed that such a preheating of the crystalline YAP: Bi detectors immediately after the irradiation removes no more than 5% of the accumulated OSL signal. For storage times longer than one week, such preheating is not mandatory, as the TSL peak at 75 °C completely fades by this time. It is worth noting that fading of the BeO Thermalox^®^995 detectors was reported to be up to 10% in 3 months^56^. Extraordinary large fading of the ceramic YAP: Bi detectors is most probably caused by large surface of grain boundaries that is an integral part of the nanocrystalline ceramics. In this way, the crystalline YAP: Bi detectors with low thermal fading appear much more preferable than the YAP: Bi ceramics.

**Fig. 11:**
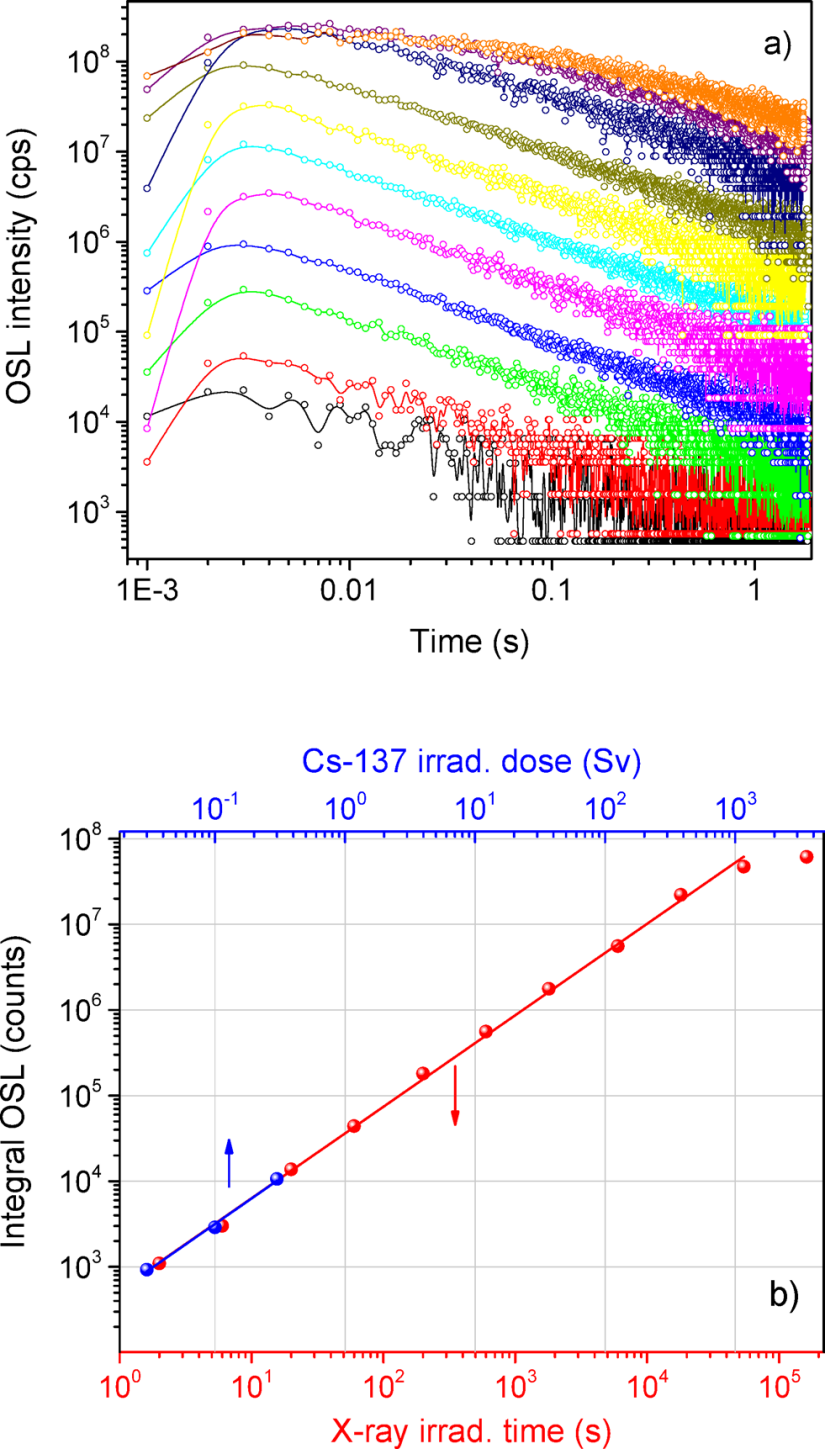
CW-OSL curves after different doses of X-rays (**a**) and the corresponding dose dependence (**b**) of the crystalline YAP: Bi detectors after X-ray (130 kV, 30 µA) and Cs-137 exposure. The solid lines represent the linear fit of the experimental points. Each experimental point was averaged after 2–3 measurements, the typical errors, estimated as the maximum deviation from the mean values, fit the point sizes.

Figure 11a shows the CW-OSL curves recorded for the crystalline YAP: Bi detectors after different doses of X-rays. The higher doses shown here were recorded using signal attenuation by the neutral density filters OD1, OD2 and OD3. It is worth noting that the CW-OSL curves have the same shape before the OSL output starts to saturate. As the OSL output begins to saturate at sufficiently high doses, the CW-OSL decay curves change their slope and become longer. The corresponding dose dependence after X-rays (derived from the data shown in Fig. 11a) by using the integral OSL output in the time range from 0 to 1 s is shown in red in Fig. 11b. The points after Cs-137 exposure corresponding to the data shown in Fig. 9 are shown in blue in Fig. 11b. The results shown in Fig. 11b confirm a perfect linearity of the OSL response of the crystalline YAP: Bi detectors with a slope coefficient equal to one over a wide range of doses. Combining of the data for X-rays and Cs-137 allows us to estimate the saturation doses to be about 1 kSv. Such a wide linearity range is a merit of the YAP crystalline host, previously confirmed for YAP: Mn^30^, which explores the intrinsic electron traps related with cation antisites^47,49^. As mentioned above, by using a more sensitive detection system it is likely to extend this range towards smaller doses.

## Conclusions and outlooks

The new high-*Z* storage phosphor YAlO_3_:Bi^3+^ (YAP: Bi) was studied for potential application in OSL dosimetry in tandem with known tissue equivalent BeO Thermalox^®^995 ceramic detectors. Two kinds of YAP: Bi samples have been prepared. The first are detectors cut from a boule of crystal grown by the Czochralski technique. The second are detectors cut from a pellet of the high-density ceramics pressed by the HPHT technique from nanocrystalline powder derived from sol-gel synthesis. The emission and dosimetric properties of the YAP: Bi detectors were explored and compared with YAP: Mn^2+^ detectors studied previously as well as with respect to its joint use with the BeO detectors in tandem.

The YAP: Bi OSL detectors are free from the drawbacks of the YAP: Mn^2+^ OSL detectors, i.e. they have no noticeable afterglow, the stimulation light doesn’t excite any photoluminescence, and the OSL decay occurs even faster than of BeO ceramics. The main feature of the YAP: Bi detectors is their UV emission at about 325 nm and the possibility of optical stimulation by blue light, which, together with above mentioned advantages allows them to be read out in the same readers used for the BeO detectors. The studied YAP: Bi detectors were found to have comparable OSL output with the BeO Thermalox^®^995 chips of comparable size. This, together with the similar registration times in CW-OSL mode, suggests that the YAP: Bi is more preferable and convenient to use in a tandem OSL dosimeter together with BeO Thermalox^®^995 chips. The sensitivity of the YAP: Bi OSL detectors could be further enhanced if the concentration of Bi^3+^ in the single crystal could be increased or the optical transparency of the YAP: Bi ceramics could be increased to at least the level of translucency. The YAP: Bi nanocrystalline ceramic detectors studied here suffer from strong thermal fading, which makes their use impractical, at least until technological methods to modify this property of ceramics are found. The modification of the grain structure of the YAP: Bi ceramics, which can also improve its optical transparency, is a main challenge in improving properties of the material for dosimetry applications.

At the same time the single crystalline YAP: Bi OSL detectors already confirm low thermal fading, sensitivity comparable to BeO and an extremely wide linearity range of dose response making them ideal as UV-emitting high-Z detectors that can be used alone or in tandem with BeO detectors.

In order to further increase the Z value of YAP: Bi OSL detectors, the modification of the host lattice with heavier rare earths, e.g. the creation of (Y, Lu)AlO_3_ solid solution as a host for Bi^3+^ activation, should be considered.

## Data Availability

All measurement data supporting the findings of this study are included in the article. Additional raw data are available from the corresponding author upon reasonable request.

## References

[1] Bøtter-Jensen, L., McKeever, S. W. S. & Wintle, A. G. Optically stimulated luminescence dosimetry. *Optically stimulated luminescence dosimetry (Elsevier)*. 10.1016/B978-0-444-50684-9.X5077-6 (2003).

[2] Olko, P. Advantages and disadvantages of luminescence dosimetry. *Radiat. Meas.* **45**, 506–511. 10.1016/J.RADMEAS.2010.01.016 (2010).

[3] Yukihara, E. G. & McKeever, S. W. S. *Optically Stimulated Luminescence: Fundamentals and Applications. Optically Stimulated Luminescence: Fundamentals and Applications* (Wiley Blackwell, 2011). 10.1002/9780470977064.

[4] Chen, R. & Pagonis, V. *Advances in Physics and Applications of Optically and Thermally Stimulated Luminescence*. (World Scientific Publishing Co., 2019). 10.1142/Q0172.

[5] McKeever, S. W. S. *A Course in Luminescence Measurements and Analyses for Radiation Dosimetry* (Wiley, 2022).

[6] McKeever, S. W. S. Optically stimulated luminescence: A brief overview. *Radiat. Meas.* **46**, 1336–1341. 10.1016/J.RADMEAS.2011.02.016 (2011).

[7] Yukihara, E. G., McKeever, S. W. S. & Akselrod, M. S. State of art: optically stimulated luminescence dosimetry – Frontiers of future research. *Radiat. Meas.* **71**, 15–24. 10.1016/J.RADMEAS.2014.03.023 (2014).

[8] Yukihara, E. G. et al. Luminescence dosimetry. *Nat. Rev. Methods Primers* 2, 1–21. 10.1038/s43586-022-00102-0 (2022).

[9] Oliveira, L. C., Yukihara, E. G. & Baffa, O. MgO:Li,Ce,Sm as a high-sensitivity material for Optically Stimulated Luminescence dosimetry. *Sci. Rep.* **6**(1), 1–12. 10.1038/srep24348 (2016). 10.1038/srep24348 PMC4830961 27076349

[10] Marczewska, B., Bilski, P., Wróbel, D. & Kłosowski, M. Investigations of OSL properties of LiMgPO_4_:Tb,B based dosimeters. *Radiat. Meas.* **90**, 265–268. 10.1016/J.RADMEAS.2016.02.004 (2016).

[11] Gieszczyk, W., Kulig, D., Bilski, P., Marczewska, B. & Kłosowski, M. Analysis of TL and OSL kinetics in lithium magnesium phosphate crystals. *Radiat. Meas.* **106**, 100–106. 10.1016/J.RADMEAS.2017.04.008 (2017).

[12] Nakauchi, D., Okada, G., Koshimizu, M. & Yanagida, T. Storage luminescence and scintillation properties of Eu-doped SrAl_2_O_4_ crystals. *J. Lumin.* **176**, 342–346. 10.1016/J.JLUMIN.2016.04.008 (2016).

[13] Luchechko, A. et al. Afterglow, TL and OSL properties of Mn^2+^-doped ZnGa_2_O_4_ phosphor. *Sci. Rep.* **9**, 1–8. 10.1038/s41598-019-45869-7 (2019). 10.1038/s41598-019-45869-7 PMC6606564 31266967

[14] Jensen, M. L. et al. Optically stimulated luminescence in state-of-the-art LYSO:Ce scintillators enables high spatial resolution 3D dose imaging. *Sci. Rep.* **12**, 1–11. 10.1038/s41598-022-12255-9 (2022). 10.1038/s41598-022-12255-9 PMC9117671 35585168

[15] França, L. V. S., Müller, E., Yukihara, E. G. & Baffa, O. A. Tb and ag co-doped Borate compound forms a high sensitive X-ray, gamma-ray and neutron luminescence dosimeter. *J. Mater. Chem. C Mater.* **11**, 4444–4455. 10.1039/D3TC00223C (2023).

[16] Vasconcelos, D. A. A., Souza, M. L., Silveira, I. S., Zanotto, E. D. & Caldas, L. V. E. Ce-doped magnesium borate glass-ceramic for optically stimulated luminescence dosimetry. *Ceram. Int.* **50**, 48988–48994. 10.1016/J.CERAMINT.2024.09.231 (2024).

[17] Ozdemir, A. et al. Luminescence properties of SrB_6_O_10_:Ce,Gd phosphor for applications in OSL dosimetry and lighting. *J. Lumin.* **273**, 120668. 10.1016/J.JLUMIN.2024.120668 (2024).

[18] Soni, A., Mishra, D. R. & Sapra, B. K. Investigation of OSL and thermally assisted OSL in Al_2_O_3_:Mg,Y phosphor. *Radiat. Phys. Chem.* **229**, 112419. 10.1016/J.RADPHYSCHEM.2024.112419 (2025).

[19] Zhang, S. et al. NaMgF_3_:Eu with improved OSL properties prepared by a simple solid-state method. *Appl. Phys. Mater. Sci. Process.* **131**, 1–10. 10.1007/S00339-025-08290-8 (2025).

[20] Altunal, V. Synthesis and luminescence characterizations of ag and Na doped LiAlO_2_ for OSL dosimetry. *J. Alloys Compd.* **1010**, 177920. 10.1016/J.JALLCOM.2024.177920 (2025).

[21] Yuan, L. et al. Optically stimulated luminescence phosphors: principles, applications, and prospects. *Laser Photon Rev.* **14**, 2000123. 10.1002/LPOR.202000123 (2020).

[22] Nanto, H. & Okada, G. Optically stimulated luminescence dosimeters: principles, phosphors and applications. *Jpn J. Appl. Phys.* **62**, 010505. 10.35848/1347-4065/AC9106 (2022).

[23] McKeever, S. W. S., Akselrod, M. S. & Markey, B. G. Pulsed optically stimulated luminescence dosimetry using Alpha-Al_2_O_3_:C. *Radiat. Prot. Dosimetry*. **65**, 267–272. 10.1093/OXFORDJOURNALS.RPD.A031639 (1996).

[24] Yukihara, E. G. A review on the OSL of BeO in light of recent discoveries: the missing piece of the puzzle? *Radiat. Meas.* **134**, 106291. 10.1016/J.RADMEAS.2020.106291 (2020).

[25] International Commission on Radiation Units and Measurement. *Report 44: tissue substitutes in radiation dosimetry and measurement*. *Journal ICRU* vol. os25 (1988).

[26] Leblans, P., Vandenbroucke, D. & Willems, P. Storage phosphors for medical imaging. *Materials*. **4**, 1034–1086. 10.3390/MA4061034 (2011). 10.3390/ma4061034 PMC5448636 28879966

[27] Liu, J. et al. Imaging and therapeutic applications of persistent luminescence nanomaterials. *Adv. Drug Deliv Rev.* **138**, 193–210. 10.1016/J.ADDR.2018.10.015 (2019). 30414492 10.1016/j.addr.2018.10.015

[28] Ou, X. et al. Recent development in X-ray imaging technology: future and challenges. *Research* **2021**10.34133/2021/9892152 (2021). 10.34133/2021/9892152 PMC8724686 35028585

[29] Michail, C. et al. Phosphors and scintillators in biomedical imaging. *Cryst. (Basel)*. **14**, 169. 10.3390/CRYST14020169 (2024).

[30] Zhydachevskii, Y., Suchocki, A., Berkowski, M., Bilski, P. & Warchol, S. Characterization of YAlO_3_:Mn^2+^ thermoluminescent detectors. *Radiat. Meas.* **45**, 516–518. 10.1016/J.RADMEAS.2009.12.035 (2010).

[31] Zhydachevskii, Y. et al. Energy response of the TL detectors based on YAlO_3_:Mn crystals. *Radiat. Meas.* **90**, 262–264. 10.1016/J.RADMEAS.2015.12.001 (2016).

[32] Zhydachevskii, Y. et al. Time-resolved OSL studies of YAlO_3_:Mn^2+^ crystals. *Radiat. Meas.* **94**, 18–22. 10.1016/J.RADMEAS.2016.08.007 (2016).

[33] Ubizskii, S. et al. A role of afterglow in optically stimulated luminescence of YAP:Mn. **141**, 379–385. 10.12693/APhysPolA.141.379 (2022).

[34] Kenney, G. N. & Cameron, J. R. *X-Ray Beam Quality Measurements Utilizing TL Dosimeters*. (1963).

[35] Gorbics, S. G. & Attix, F. H. LiF and CaF_2_:Mn thermoluminescent dosimeters in tandem. *Int. J. Appl. Radiat. Isot.* **19**, 81–84. 10.1016/0020-708X(68)90074-4 (1968). 5639088 10.1016/0020-708x(68)90074-4

[36] Dixon, R. L. & Watts, F. C. The use of BaF_2_ thermoluminescence in determining radiation quality. *Phys. Med. Biol.* **17**, 81. 10.1088/0031-9155/17/1/009 (1972). 5071505 10.1088/0031-9155/17/1/009

[37] Spurny, Z. Some new materials for TLD. *Nucl. Instr Meth*. **175**, 71–73 (1980).

[38] Da Rosa, L. A. R. & Nette, H. P. Thermoluminescent dosimeters for exposure assessment in gamma or X radiation fields with unknown spectral distribution. *Int. J. Rad Appl. Instrum. A*. **39**, 191–197. 10.1016/0883-2889(88)90171-2 (1988).

[39] Chumak, V. et al. Passive system characterizing the spectral composition of high dose rate workplace fields: potential application of high Z OSL phosphors. *Radiat. Meas.* **106**, 638–643. 10.1016/J.RADMEAS.2017.07.008 (2017).

[40] Ubizskii, S., Poshyvak, O. & Zhydachevskii, Y. Analysis of the radioisotopes recognition possibility by means of the absorbed dose measurement with dosimetric detectors of different density. *Inform. Communication Technol. Electron. Eng.* **3**, 154–162. 10.23939/ICTEE2023.01.154 (2023).

[41] Ubizskii, S., Pawlowska, Z., Stasiv, V., Poshyvak, O. & Zhydachevskyy, Y. The High-Z dosimetric phosphor for the urgent emergency dosimetry. In *Conf. Proc. – 2024 IEEE 17th Int. Conf. Adv. Trends Radioelectronics Telecommunications Comput. Eng. TCSET 2024*, 349–354 10.1109/TCSET64720.2024.10755616 (2024).

[42] Yukihara, E. G., Bos, A. J. J., Bilski, P. & McKeever, S. W. The quest for new thermoluminescence and optically stimulated luminescence materials: needs, strategies and pitfalls. *Radiat. Meas.* **158**, 106846. 10.1016/J.RADMEAS.2022.106846 (2022).

[43] Ubizskii, S., Afanassyev, D., Zhydachevskii, Y., Rabyk, V. & Luchechko, A. Concept development of a portable reader for personal dosimetry based on the OSL in yap:mn. In *Proc. – 15th Int. Conf. Adv. Trends Radioelectronics Telecommunications Comput. Eng. TCSET 2020*, 952–956 10.1109/TCSET49122.2020.235578 (2020).

[44] Krasnikov, A. et al. Time-resolved photoluminescence and excited state structure of Bi^3+^ center in YAlO_3_. *Opt. Mater. (Amst)*. **36**, 1705–1708. 10.1016/J.OPTMAT.2014.01.030 (2014).

[45] Baran, M., Stasiv, V., Vasylechko, L., Zazubovich, S. & Zhydachevskyy, Y. Thermally stimulated luminescence of UV-irradiated YAlO_3_:Bi perovskite. *J. Lumin.* **276**, 120875. 10.1016/J.JLUMIN.2024.120875 (2024).

[46] Baran, M. et al. Bi^3+^ - doped garnets as possible ultraviolet persistent phosphors. *Opt. Mater. (Amst)*. **137**, 113584. 10.1016/J.OPTMAT.2023.113584 (2023).

[47] Przybylińska, H. et al. Electron paramagnetic resonance and optical studies of thermoluminescence processes in Mn-Doped YAlO_3_ single crystals. *J. Phys. Chem. C*. **126**, 743–753. 10.1021/acs.jpcc.1c08997 (2022).

[48] Zhydachevskyy, Y. et al. Band gap engineering and trap depths of intrinsic point defects in RAlO_3_ (R = Y, la, gd, yb, Lu) perovskites. *J. Phys. Chem. C*. **125**, 26698–26710. 10.1021/acs.jpcc.1c06573 (2021). 10.1021/acs.jpcc.1c06573 PMC8672454 34925675

[49] Zhydachevskii, Y. et al. Technological approaches for improving thermoluminescent properties of the Czochralski-grown YAlO_3_:Mn crystals. *J. Cryst. Growth*. **310**, 3219–3223. 10.1016/J.JCRYSGRO.2008.03.043 (2008).

[50] Sugak, D. et al. Growth and induced color centers in YAlO_3_–Nd single crystals. *Phys. Status Solidi (a)*. **184** (2001).

[51] Matkovskii, A. O. et al. Growth and properties of YAlO_3_:Tm single crystals for 2-µm laser operation. *J. Cryst. Growth*. **241**, 455–462. 10.1016/S0022-0248(02)01245-9 (2002).

[52] Prakasam, M. et al. Ultrahigh pressure SPS (HP-SPS) as new syntheses and exploration tool in materials science. *Spark Plasma Sintering: Curr. Status New. Dev. Challenges*. 201–218. 10.1016/B978-0-12-817744-0.00009-X (2019).

[53] Yavetskiy, R. P. et al. Y_3_Al_5_O_12_ translucent nanostructured ceramics—Obtaining and optical properties. *Ceram. Int.* **37**, 2477–2484. 10.1016/J.CERAMINT.2011.03.041 (2011).

[54] Diehl, R. & Brandt, G. Crystal structure refinement of YAlO_3_, a promising laser material. *Mater. Res. Bull.* **10**, 85–90. 10.1016/0025-5408(75)90125-7 (1975).

[55] Tochilin, E., Goldstein, N. & Miller, W. G. Beryllium oxide as a thermoluminescent dosimeter. *Health Phys.* **16** (1969). 10.1097/00004032-196901000-00001 5766055

[56] Sommer, M. & Henniger, J. Investigation of a BeO-based optically stimulated luminescence dosemeter. *Radiat. Prot. Dosimetry*. **119**, 394–397. 10.1093/RPD/NCI626 (2006). 16735572 10.1093/rpd/nci626

[57] Zhydachevskyy, Y. et al. Thermally induced fading of Mn-doped YAP nanoceramics. *J. Phys. Conf. Ser.* **987**, 012009. 10.1088/1742-6596/987/1/012009 (2018).

